# The Epigenetics Dilemma

**DOI:** 10.3390/genes11010023

**Published:** 2019-12-23

**Authors:** Christoph Grunau, Jérémy Le Luyer, Martin Laporte, Dominique Joly

**Affiliations:** 1 Univ. Perpignan Via Domitia, IHPE UMR 5244, CNRS, IFREMER, Univ. Montpellier, F-66860 Perpignan, France; 2 IFREMER/Centre du Pacifique, Département Ressources Biologiques Environnement, Unité Ressources Marines en Polynésie Française, UMR 241 Écosystèmes Insulaires Océaniens, BP 49, 98719 Taravao, Tahiti, Polynésie Française; jeremy.le.luyer@ifremer.fr; 3 Institut de Biologie Intégrative et des Systèmes (IBIS), Deépartement de Biologie, Université Laval, Pavillon Charles-Eugène-Marchand, Quebec, QC G1V 0A6, Canada; uni.mlaporte@gmail.com; 4 Laboratoire Evolution, Génomes Comportement, Ecologie, UMR 9191, CNRS, IRD, Université Paris Saclay, Avenue de la Terrasse, 91198 Gif sur Yvette, France; dominique.joly@cnrs-dir.fr

This special issue of Genes demonstrates clearly that research in epigenetics has proceeded at a very rapid pace in the last decade. A wide range of techniques is available to those who endeavor studies in epigenetics and as long as there is a research budget, there remains today very few technical constraints. It is, for instance, conceivable, as demonstrated by Liu et al. in this special issue [1], to practically start from scratch to sequence and assemble the genome of any species, establish the epigenome, and perform integrative comparative approaches on both within the framework of a single publication. This would have been inconceivable only a couple of years ago. As a consequence, results presented in this issue and elsewhere make clear that this is a time of opportunity for epigenetics as its contours and impact are traced more and more clearly: the epigenome is demonstrated to be very plastic, it changes during development [2], but also, and in a different way, when exposed to external environmental cues. These changes occur sometimes within generations [3] and, in other cases, epigenetic plasticity occurs through generations [4,5], conveying parental effects of very different type (e.g., parental diet or hatchery environment).

However, this plastic character of the epigenome makes it also difficult to draw general conclusions on how every epigenotype, genotype and phenotype are interrelated. In this issue, it is shown that phenotypic, and thus potentially gene expression changes, can precede epigenetic changes [3], or that they occur after the initial environmental stimulus [4,5]. It is also shown that epigenetic modifications can be associated with the magnitude of change in reaction norms of some but not all phenotypic traits [6]. In addition, even when considering the same bearer of epigenetic information (e.g., DNA methylation), different organisms show different types of DNA methylation (e.g., [1,2,3,4,5,6]) with potentially different types of phenotypic effects. Nevertheless, epigenetic changes do not occur randomly along the genome. In the two examples presented in this issue, environmentally induced epimutations were clearly enriched in the pathways of alert information and signaling: between 40% [5] and 66% [4] of differentially methylated regions (DMR) occurred in genes associated with signal transduction. This suggests that DMR are involved in the management of enduring and adequate stress response information.

The current special issue of Genes thus reflects a peculiar situation for epigenetics research. We can accumulate, through hard work but eventually easily, large epigenomic data on a wide range of model and non-model organisms. We see clearly that environmental cues, especially stress, lead to specific changes in the epigenome that are accompanied by phenotypic modifications. We also see the relationship between epigenetic modifications and genomic plasticity (a large body of evidence in this issue in the reviews of [7,8]). However, the dilemma is that it is still unclear for most species how exactly phenotype and epigenotype are linked.

There are several reasons for this. First, it is the very definition of epigenetics (considered a “heritable but reversible changes in gene function”; paraphrased as e.g. “regulation of transcription” used in [2], “gene activation” by Gavery et al. [4], and “gene expression” in [6]) that makes a clear and unambiguous claim of a link between epigenetics and gene function, i.e., the (molecular) phenotypes. Such a claim is absent from definitions of genetics (“Genetics is the study of heredity”; e.g., [9]) thus orphaning those changes in chromatin structure that do not lead to detectable changes in gene function. One is right to wonder whether the current definition of epigenetics is the best or if it should it be amended.

Second, the dynamics of epigenetic plasticity are often very much higher than that of the genome, i.e., in observable time-span epigenetic information changes, resulting from, accompanying, or leading to phenotypic changes. To capture the full complexity of this relationship and establish causality, it is indispensable to produce longitudinal data and analyze time series. Once such relations are clearly established in one species, the phenotypic expression of epigenetic information might still be different in other species. Thus, we need to accumulate more data from non-model species to provide testable hypothesis on the genotype–epigenotype–phenotype relationship and to establish predictive models for the different epigenome types.

Third, the “dark matter” of the genome (intergenic regions, transposable elements, and other repetitive DNA) are often only considered when they influence gene transcription. This might be too narrow a view. The sheer amount of “non-coding” RNA, the absence of transcription information for the majority of it and anecdotic evidence for correlation to phenotypic changes justifies a broader interest into this compartment of the genome and they should systematically be included into (epi) genomics studies.

Consequently, we believe that, if one would have to decide on research priorities for the time to come, it would certainly be useful (i) to define and redefine what epigenetic actually means and how it might be differently defined in different contexts. If the close relationship to the phenotype is maintained then (ii) it will be necessary to develop conceptual and mathematical tools for integrating genetic, epigenetic, and phenotypic variation (which would therefore need to be produced also concomitantly and over time series) in genes and repetitive elements. In addition, functional epigenomics approaches in which targeted epimutations are produced in a wide range of organisms to investigate resulting phenotypes will provide a promising venue to unambiguously link epigenotype and phenotype and to solve the current epigenetics dilemma.
